# Items

**Published:** 1883-01

**Authors:** 


### Items.

METEOROLOGICAL DATA.—For the month of Nov., 1882. H. C. Smyth, Observer, U. S. Signal Station, Atlanta, Ga.

| | | |
|---|---|---|
| BAROMETER | —Mean | 30.205 |
| | Highest | 30.504 on 30th. |
| | Lowest | 29.865 on 20th. |
| | Range | .639 |
| THERMOMETER | —Mean | 50.6 |
| | Highest | 80.5 on 2d. |
| | Lowest | 28.7 on 30th. |
| | Range | 51.8 |
| | Greatest Daily Range | 27.0 on 13th. |
| | Least Daily Range | 7.0 on 19th. |
| Humidity | —Mean | 66.0 |
| Dew-point | —Mean | 38.5 |
| Rainfall | —Total | 4.22 inches. |
| | Greatest Daily | 1.49 on 20th. |
| | Total Number of Days | 9 |
| Wind | —Prevailing Direction | W. |
| | Maximum Velocity | N. E., 37 miles. |

---

THE *Medical and Surgical Reporter* gives expression to the following sentiment:

A medical college, to do any real good, must have a number of extrinsic advantages. It requires a city of considerable size, from which to draw sufficiently varied clinical material; it must be in a locality where able men can obtain a good living from their practice, as otherwise they will not come to it as teachers; its organization must not be under the control of one man, nor must financial success be necessary to its existence. This last touches the white. It means that the corporation must be with ample resources to begin with. There ought to be a law in every State prohibiting the establishment of any medical college unless the corporators could show $100,000.00 paid up capital and an agreement to fund all profits for ten years! A poor man is generally despised, whether he deserves it or not, but a poor medical college always deserves it. No professional men have a right to band together to set on its legs an impecunious institution. The history of all such enterprises is a long list of jealousies and back-biting, degrading competition for students, wretchedly superficial instruction, deficient anatomical and clinical material, servility in lecturing, a sham of an examination, and a lot of grossly incompetent young men turned out to prey on the community as doctors.

If by accident an honest man is on the faculty and exposes this miserable history, then comes a scene of slander and recrimination, which stains the whole profession with its inkiness.

---

THE *Maryland Medical Journal* quotes the following report of the Judicial Committee of the American Medical Association, upon which the use of special signs and cards is based: "The acceptance of this honorable title" (Doctor) "is presumptive evidence to the community that the man accepting it is ready to attend practically to any and all duties which it implies. As all special practice is simply a self-imposed limitation of the duties implied in the general title of Doctor, it should be indicated, not by special or qualifying titles, such as Oculist, Gynæcologist, etc., nor by any positive setting forth of special qualifications, but by a simple, honest notice appended to the ordinary card of the general practitioner, saying, 'practice limited to diseases of the eye or ear,' or 'diseases peculiar to women,' or 'to midwifery exclusively,' as the case may be."

The *Louisville Medical News* quotes the following paragraph from the introductory lecture of Jonathan Hutchinson at the London Hospital:

"If now I were to sum up in one sentence what I have been enforcing it would be this: The secret of all noble life lies in belief, and the characteristic of all noble minds is the vigor with which they believe that which is true. Try to attain belief in the reality of all things, so shall you never want for motives, so shall you be able to live and work without hurry and without sloth. Finally, permit me to commend to you this formula: Prize strength, love the beautiful, practice self-denial, and be patient."

---

The suit of Brighton, England, against the *London Lancet* for alleged libelous publication concerning the insalubrious condition of that famous resort, has been withdrawn. Meanwhile it is hoped that the causes of the strictures have been removed. We thought at the time the suit was threatened that an enterprising corporation could find better use for twenty-five thousand dollars than its expenditure in the prosecution of a public journal which was only endeavoring to do its whole duty in sounding, no doubt, a justifiable warning.

---

We learn from the Hot Springs (Arkansas) *Daily Sentinel* that Dr. J. T. Jelks, of that place, formerly of Marietta, Ga., has accepted the chair of Surgical Diseases of the Genito-Urinary Organs, in the College of Physicians and Surgeons, Chicago, and that he will soon leave Hot Springs and take up his residence in Chicago for the purpose of entering actively upon the discharge of his new duties there.

---

Attention is directed to the fact that, according to the requirements of the last pharmacopœia, the strength of the liquid preparations of opium, excepting paragoric, is increased. Of the tincture, the deodorized tincture and the compound solution sixteem minims or twenty-five drops, represent twenty-four minims or thirty-eight drops of the pharmacopœia of 1870 and which is equivalent to one-quarter of a grain of morphine. It is important that this change should be borne in mind by prescribers and pharmacists.

---

AT a banquet given in London to the returned medical officers of the English army in Egypt, Mr. Spencer Wells, in the course of his post-prandial speech, said: "The first Jenner is immortal as a benefactor of mankind. It may not be generally known, but it is true, that the first Jenner has saved, is now saving, and will continue to save in all coming ages, more lives in one generation than were destroyed in all the wars of the first Napoleon."

---

MESSRS. J. B. LIPPINCOTT & Co. announce the early appearance of the Fifteenth Edition of the United States Dispensatory, edited by Drs. H. C. Wood, Joseph P. Remington and Samuel P. Sadler. This book is well known to the profession, and the publishers promise that its relation to the new Pharmacopœia will be fully maintained, and that the latter work, in all its parts, will be freely expounded and discussed.

---

ACCORDING to a recent declaration of the *Medical Press and Circular*, there are many Continental and a few American and Colonial licensing institutions which might be admitted within the pale without hesitation, but they are numerically insignificant in comparison to the multitude of "diploma mills" in America and elsewhere, "run," as the phrase goes, by needy, avaricious, unprincipled, and obscure medical men.

---

THE Board of Health of Boston has prohibited public funerals in the case of persons dead from small-pox, diphtheria, scarlet fever and typhoid fever. The remains are required to be placed in a properly sealed receptacle as soon as possible after death and the apartments, occupied during the last sickness, must be fumigated and disinfected.

---

DR. JOHN E. BACON, of Columbus, and Dr. Geo. F.

Cooper, of Americus, two well-known and highly esteemed physicians, died recently in their respective homes. Both were well advanced in years, and both had been faithful in their day and generation. No doubt they are rejoicing in the reward of the just.

---

If every community in which small-pox appears would do its whole duty, the country would soon escape the vexatious annoyance occasioned by the too frequent outcropping of this preventable disease.

---

Dr. Oliver Wendell Holmes, now in his seventy-fourth year, has resigned the chair of anatomy in the medical department of Harvard University, which he has occupied for thirty-six years.

---

Is the gustatory nerve of the fowl distributed to the lining membrane of the gizzard? If not, what enjoyment can be obtained when they feed on grain, and what is the incentive to eat it?

---

Does the *Columbus* (Ohio) *Medical and Surgical Journal* think it is the correct thing to republish articles from other journals without giving due credit? We ask for information.

---

Always avoid vague approximations where exact estimates are possible; *about so many*, *about so much*, instead of the precise number and quality.—*Holmes.*

---

Dr. Robert Battey has been notified of his election to an honorary fellowship in the Obstetrical Society of Edinburg.

---

Victim, of the bungling arts and cruel devices of shallow brains, thy name is woman.

---

Some medical men and some medical journals are nothing if they are not foreign.

---

The secret of a good judgment lies in the habit of thinking correctly.

